# Epidemiological Characteristics of Upper Respiratory Tract Pathogens in Children in Guangdong, China

**DOI:** 10.1111/crj.70011

**Published:** 2024-10-05

**Authors:** Qianwen Zhao, Peifeng Ke, Liangshan Hu, Changhong Jiang, Rong Su, Weifeng Lv, Qixin Li, Lingxiao Jiang, Donglin Cao

**Affiliations:** ^1^ Division of Laboratory Medicine, Zhujiang Hospital Southern Medical University Guangzhou Guangdong China; ^2^ Department of Laboratory Medicine The Second Affiliated Hospital of Guangzhou University of Chinese Medicine Guangzhou Guangdong China; ^3^ Guangdong Provincial key Laboratory of Research on Emergency in TCM Guangzhou Guangdong China; ^4^ Department of Laboratory Medicine The Affiliated Guangdong Second Provincial General Hospital of Jinan University Guangzhou Guangdong China; ^5^ Department of Laboratory Medicine Foshan Hospital of Traditional Chinese Medicine Foshan Guangdong China; ^6^ Department of Clinical Laboratory The First People's Hospital of Foshan Foshan Guangdong China

**Keywords:** multiplex probe amplification PCR, pediatric respiratory infections, seasonal virus variations, upper respiratory tract viruses, virus epidemiology in Guangdong

## Abstract

**Objective:**

Researches on the epidemiology of various respiratory pathogens at multiple testing points in the pediatric population are limited, and these are crucial for the prevention of respiratory tract infections in children.

**Methods:**

We obtained 1788 upper respiratory tract swabs from children exhibiting symptoms of respiratory infection (notably fever with a body temperature exceeding 38.5°C) across five hospitals in Guangdong between November 2020 and June 2022. We used the multiplex probe amplification (MPA) PCR testing to identify 11 respiratory viruses and subsequently analyzed the prevalence characteristics of these pathogens among febrile children in hospitals.

**Results:**

The overall detection rate of the pathogens was 58.1% (1039/1788). Human rhinovirus (HRV) exhibited the highest detection rate at 19.0% (339/1788), succeeded by human parainfluenza virus (HPIV), human adenovirus (HAdV), and respiratory syncytial virus (RSV). The positivity and coinfection rates were higher in children aged 5 years and below compared to those above 5 years. Moreover, a distinct pathogen spectrum was observed across different age groups. Hospitalized patients demonstrated a significantly higher positivity and coinfection rate compared to outpatients. During COVID‐2019, RSV appeared a counter‐seasonal trend.

**Conclusion:**

Respiratory viral infections in children display distinct characteristics concerning age, hospitalization status, and seasonality. Children under the age of 5 and minor patients admitted to hospitals at least be tested for RSV, HRV, HPIV, and HAdV. The epidemiological patterns of RSV in the post‐epidemic period require ongoing surveillance.

## Graphical abstract

Prevalence of pediatric respiratory viruses in Guangdong: Study of 1788 children reveals a 58.1% pathogen detection rate, with human rhinovirus most prevalent. Highlights need for targeted diagnostic strategies in pediatric respiratory infections. Impact of COVID‐19 on seasonal virus patterns: Observes a counter‐seasonal trend in respiratory viruses, particularly RSV, during the pandemic. Highlights how public health measures have influenced typical viral epidemiological patterns. Diagnostic advancements with multiplex probe amplification PCR: utilization of advanced diagnostics like multiplex probe amplification PCR demonstrates its efficacy in detecting multiple pathogens, influencing treatment strategies and infection control in pediatric care.

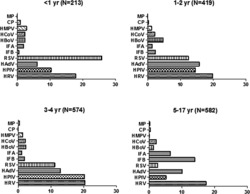

## 1 Introduction

Respiratory tract infections (RTIs) represent the leading cause for pediatric medical consultations, with viruses being predominant etiological agents during childhood. Studies indicate that respiratory viruses account for up to 90% of lower RTIs during infancy, underscoring the global significance of pediatric respiratory viral infections [1]. The primary viruses associated with respiratory infections in children encompass respiratory syncytial virus (RSV), influenza virus (IFV), human rhinovirus (HRV), human adenovirus (HAdV), human parainfluenza virus (HPIV), human metapneumovirus (HMPV), and human coronavirus (HCoV), among others. Notably, each virus exhibits distinct epidemiological profiles and clinical presentations [2, 3, 4].

Rapid pathogen identification forms the foundation for effective treatment in several key ways. Firstly, symptoms of respiratory infections in children often overlap, with common complaints such as cough, sore throat, and fever. These symptoms can be triggered by a variety of different pathogens. Pathogen identification alleviates strain on the healthcare system by minimizing unnecessary antibiotic treatments and other ineffective tests for viral infections [2] and empowers medical professionals to select the most appropriate treatment strategy [5, 6]. Secondly, in cases where children are infected with pathogens, early identification and patient isolation serve as effective measures to curtail the spread of viruses within families, schools, and communities. Swift detection is particularly crucial for highly contagious viruses, such as COVID‐2019 [7, 8]. Lastly, public health departments rely on monitoring valuable epidemiological data, including information about emerging virus strains, to aid in preventing and controlling large‐scale outbreaks. Pathogen nucleic acid testing provides readily available data to support these efforts. The rapid and precise detection of pathogens holds immense significance in these contexts.

The prevalence of pathogens varies across regions and climates, necessitating real‐time and continuous monitoring for precise prevention and control, as well as accurate diagnosis and treatment [3, 4, 9, 10]. The outbreak of COVID‐2019, prompting the implementation of various measures such as mask‐wearing, social distancing, and home isolation to curb its spread. Interestingly, these measures may have effectively mitigated other infections during the initial stages of the pandemic. The dynamics of respiratory virus transmission have indeed induced shifts in the pathogen prevalence landscape, both regionally and temporally [11, 12, 13]. This highlights the crucial need for more comprehensive and in‐depth epidemiological surveillance data. During the COVID‐19 epidemic, viral nucleic acid detection technology not only aided in clinical prognosis assessment but also enabled hospitals to adopt practical measures for infection prevention during an outbreak. This approach disrupts the transmission chain and curtails viral spread, playing a pivotal role in diagnosis and treatment advancements [14]. To explore the prevalence of common upper respiratory tract pathogens among children in Guangdong during the epidemic, we conducted a random sampling of 1788 samples collected from pediatric patients presenting with fever symptoms in five hospitals across Guangdong. Utilizing multiplex fluorescence quantitative PCR, we conducted pathogen detection through nucleic acid testing and performed statistical analysis to assess the prevalence of various pathogens.

## 2 Materials and Methods

### 2.1 Participants

From November 2020 to June 2022, this study collected data from 1788 pediatric patients (under 18 years old) who were diagnosed with fever symptoms (with a body temperature higher than 38.5°) from five hospitals in Guangdong, which included The Second Affiliated Hospital of Guangzhou University of Chinese Medicine, The Affiliated Guangdong Second Provincial General Hospital of Jinan University, The First People's Hospital of Foshan, Foshan Hospital of Traditional Chinese Medicine, and Zhujiang Hospital, Southern Medical University. The chosen period ensured complete and consistent data collection. The samples were categorized into four age groups: infants (< 1 year old), toddlers (1–2 years old), school‐age toddlers (3–4 years old), and school‐age adolescents (5–17 years old). Additionally, these samples were further divided into outpatient and inpatient groups based on the medical records of each hospital. Specifically, outpatients received medical consultation and treatment without hospital admission, whereas inpatients were admitted to the hospital for treatment.

Nasopharyngeal swabs from the patients served as experimental samples, and they were collected by specialized personnel from the respective laboratory departments. The experiments were conducted within the laboratories of each hospital's laboratory department. The patients' essential clinical information was recorded and extracted from the hospital's electronic medical record system. However, it is important to note that no personally identifiable information of the patients was exported as part of this study.

### 2.2 Methods

#### 2.2.1 Nucleic Acid Extraction

Nucleic acids were extracted using an automated nucleic acid extractor (Daan Smart 32, Guangzhou Da'an Gene Co., Ltd., Guangzhou, China), following the manufacturer's instructions. The extracted nucleic acids were stored at −80°C for preservation.

#### 2.2.2 Multiplex Probe Amplification (MPA) PCR Testing

Nucleic acids stored at −80°C were subjected to testing for multiple pathogens using commercial kits following the manufacturer's instructions. The PCR detection kit was purchased from Guangzhou Biotron Technology Co., Ltd (Guangzhou, China). The kit name and catalog number are as follows: “Respiratory 12‐item Pathogen Nucleic Acid Detection Kit (Fluorescent PCR Melting Curve Method)” with catalog number “BB5013,” including *Chlamydia pneumoniae*, *Mycoplasma pneumoniae*, adenovirus (Group B, Group C, Group E), influenza A virus (IFA) (H1N1, H3N2, H1N1 [2009], H5N1, and H7N9), human influenza B virus (IFB) (Victoria and Yamagata), parainfluenza virus (Types 1–4), rhinovirus, RSV (Type A and B), bocavirus, metapneumovirus, coronavirus (229E, HKU1, NL63, and OC43), ORF1ab gene of COVID‐19, and N gene of COVID‐19. Subtyping was not performed for adenovirus, IFA, IFB, parainfluenza virus, RSV, and coronavirus. Primer sequences are proprietary and cannot be disclosed.

#### 2.2.3 Statistical Analysis

Data collection and biostatistical analysis were performed using EXCEL and SPSS software. GraphPad Prism 5.0 software and ChatGPT 4.0 were utilized for data visualization.

## 3 Results

### 3.1 Detection of Feverish Patient

Between November 2020 and June 2022, a total of 1788 fever patient samples from five hospitals in Guangdong were analyzed. The overall proportion of patients with positive results for at least one pathogen was 58.1% (1039/1788). The male‐to‐female ratio was 1.51 (1074/714), and there was no significant difference in positivity rates between genders (*p* = 0.379) (Table 1). However, there were significant differences in virus positivity rates among the four age groups (*p* < 0.05). School‐age adolescents (5–17 years old) exhibited the lowest virus positivity rate at 53.1% (309/582), whereas the other three age groups all had positivity rates exceeding 60% (Table 1). Furthermore, a significant disparity in virus positivity rates was observed between inpatients and outpatients, with inpatients having a higher rate of 62.0% (683/1101) compared to outpatients at 51.8% (356/687) (Table 1).

**TABLE 1:** Characteristics of children with fever.

Characteristic	Virus positive	Virus negative	Proportion of positive pathogen
Sex			
Male	615	459	57.3%
Female	424	290	59.4%
*χ2*	0.793		
*p*	0.379		
Age group			
< 1 year	129	84	60.6%
1 to < 3 years	256	163	61.1%
3 to < 5 years	345	229	60.1%
5 to < 18 years	309	273	53.1%
*χ2*	**9.020**		
*p*	< 0.05*		
Case type			
Inpatients	683	418	62.0%
Outpatients	356	331	51.8%
*χ2*	**18.134**		
*p*	< 0.001*		
All	1039	749	

*Note:* The chi‐square (χ2) test was performed using SPSS. A *p* < 0.05 is indicated by * and is considered statistically significant (in bold).

### 3.2 Pathogen Etiology

**FIGURE 1:**
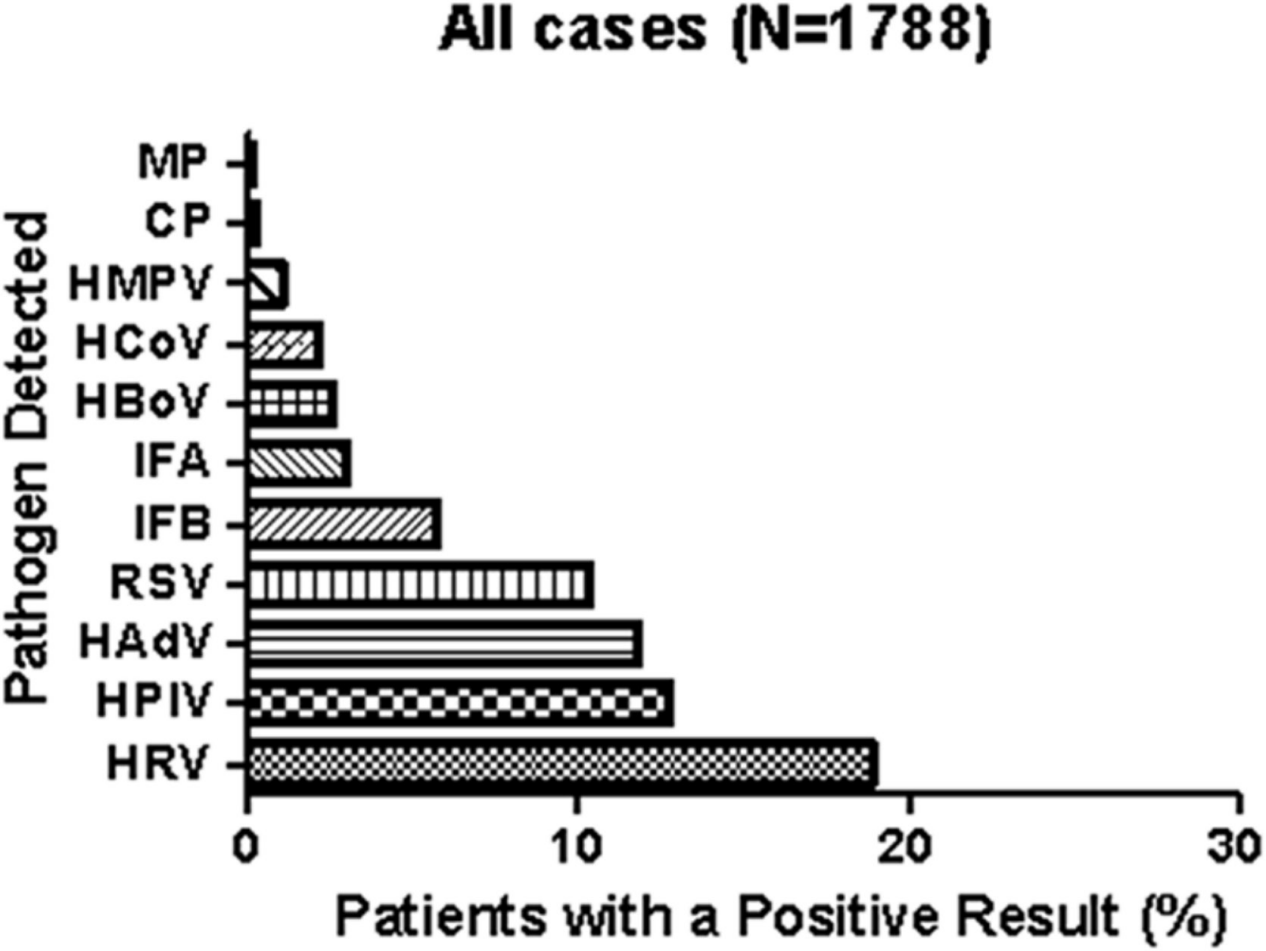
Detection of pathogens to all cases.

Among patients infected with at least one pathogen, the pathogen with the highest positive rate was HRV at 19.0% (339/1788), followed by HPIV at 12.8% (229/1788), HAdV at 11.9% (212/1788), and RSV at 10.4% (186/1788). The positive rates for other pathogens, including IFB, IFA, human bocavirus (HBoV), HCoV, HMPV, *C. pneumoniae* , and *M. pneumoniae* (MP), were 5.8%, 3.0%, 2.6%, 2.2%, 1.1%, 0.3%, and 0.2%, respectively (Figure 1).

The distribution of these pathogens varied among the four age groups. Young children under 1 year old were primarily infected with RSV, followed by HRV and HPIV.

**FIGURE 2:**
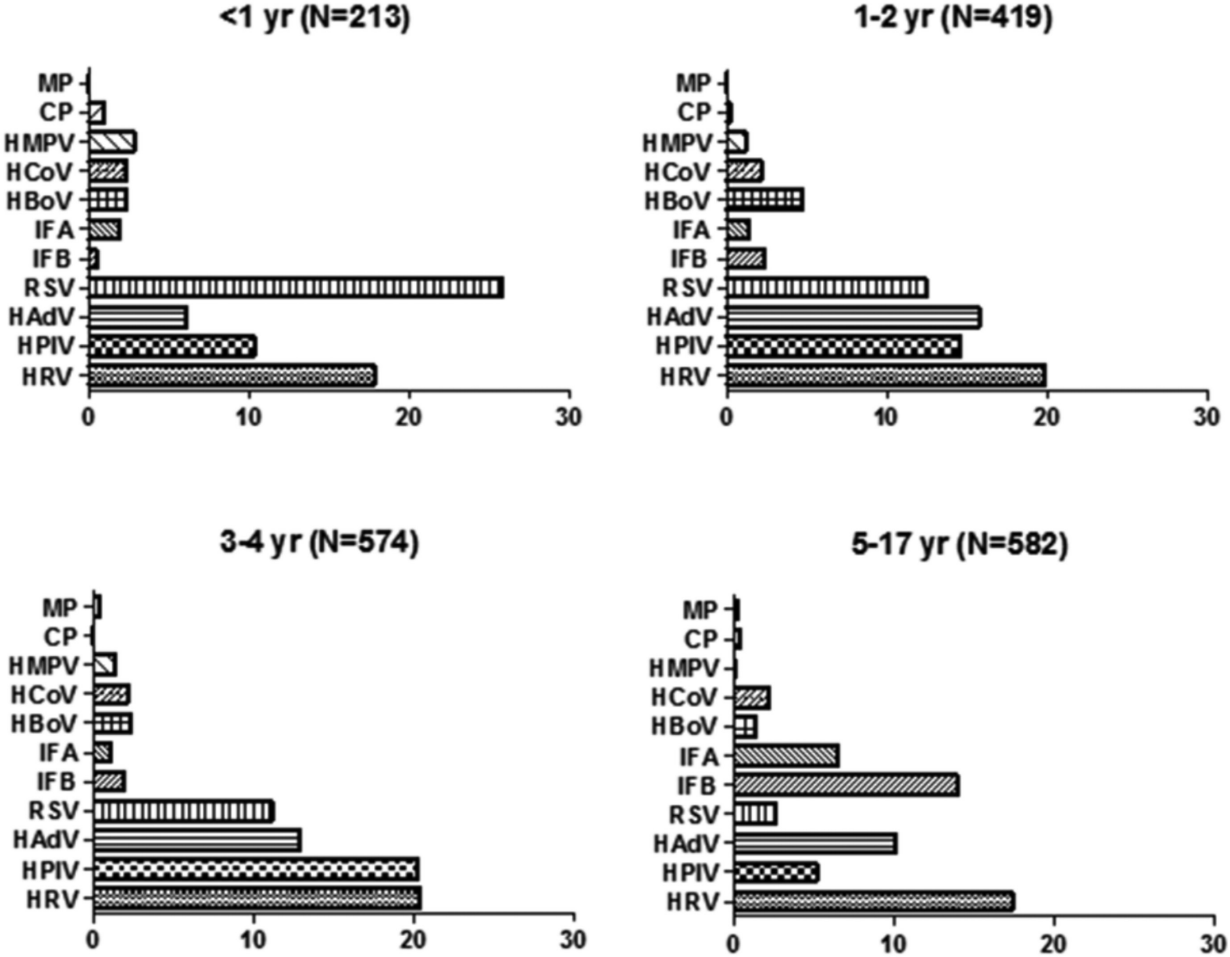
Detection of pathogens to age group.

Children aged 1–2 years were mainly infected with HRV, HAdV, and HPIV. In the 3–4 year age group, HRV, HPIV, and HAdV were the predominant pathogens, with HRV and HPIV having the highest detection rates in this age group. Among individuals aged 5–17 years, HRV, IFB, and HAdV were the primary pathogens, with a notable increase in the detection rate of IFB in this age group, second only to HRV (Figure 2).

**FIGURE 3:**
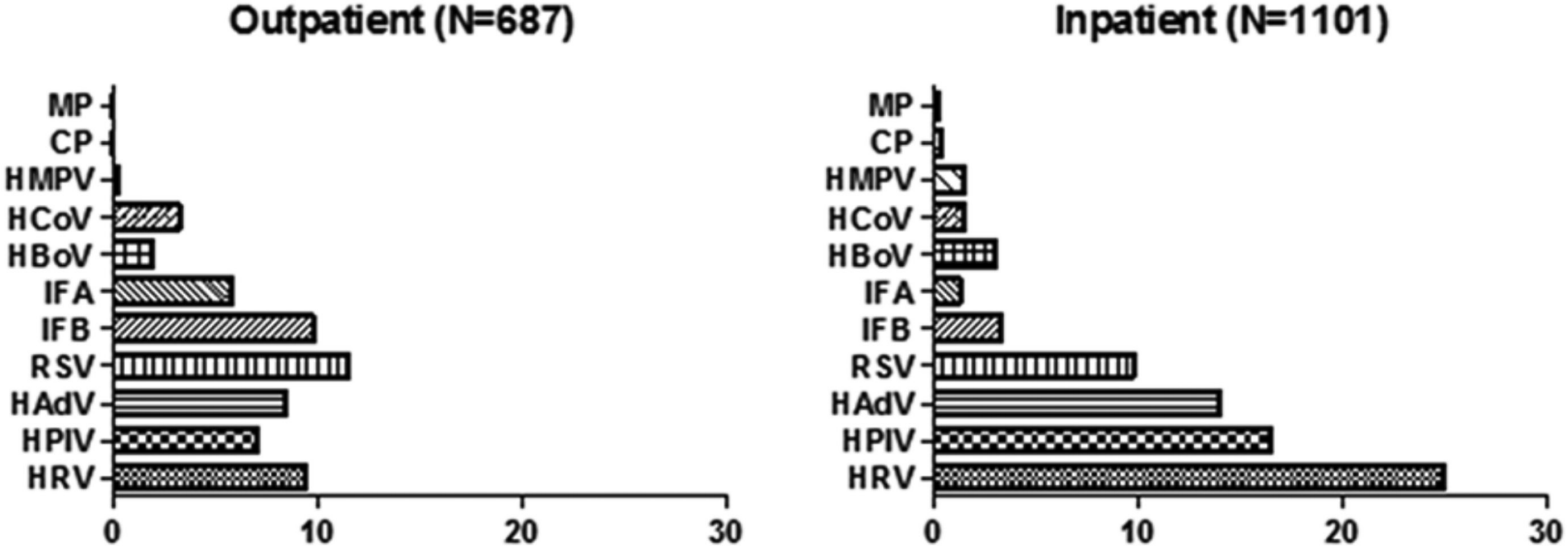
Detection of pathogens to outpatients and inpatients.

The spectrum of prevalent pathogens differed between outpatients and inpatients. Among outpatients, the pathogen with the highest positive rate was RSV, followed by IFB and HRV. Conversely, among inpatients, HRV had the highest positive rate, followed by HPIV and HAdV (Figure 3).

### 3.3 Seasonal Features

**FIGURE 4:**
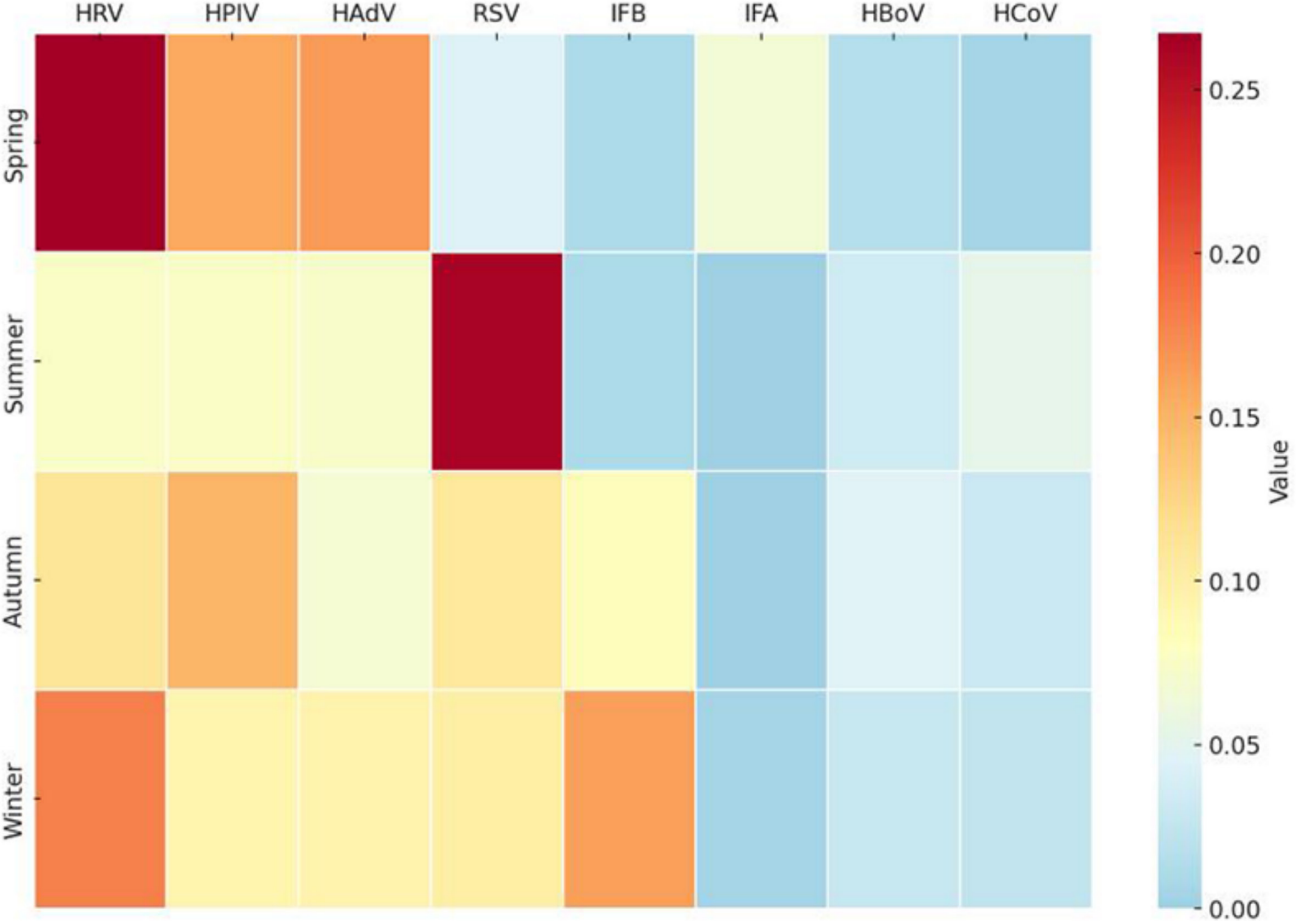
Heatmap of seasonal features for pathogens.

A chi‐square test was conducted to assess the seasonal patterns of viruses with more than 30 positive samples. The results indicated that, except for HBoV, all other viruses had *p* values less than 0.01, signifying highly significant differences in seasonal characteristics (Table 2). The heat map of positivity rates revealed that RSV was predominant during the summer months, a deviation from the usual winter peak, reflecting potentially altered transmission dynamics amidst public health interventions. In contrast, other pathogens were more prevalent in the winter or spring seasons. IFB exhibited its peak prevalence during the winter, whereas IFA was primarily prevalent in the spring (Figure 4). This visualization underscores the distinct seasonal patterns that vary significantly among the pathogens studied.

**TABLE 2:** Seasonal features for pathogen (*N* = 1788).

	HRV		HPIV		HAdV		RSV		IFB		IFA		HBoV		HCoV	
	+	−	+	−	+	−	+	−	+	−	+	−	+	−	+	−
Spring	782	209	573	124	658	130	652	34	748	8	774	52	730	12	770	4
Summer	301	23	278	23	278	22	279	79	222	3	298	0	301	10	291	16
Autumn	288	32	256	43	245	20	268	31	257	24	264	0	288	13	275	9
Winter	417	75	342	39	378	40	377	42	375	68	349	2	415	11	406	10
*χ2*	**47.125**		**23.265**		**28.654**		**73.160**		**112.579**		**53.449**		6.846		**24.564**	
*p*	< 0.001*		<v0.001*		< 0.001*		< 0.001*		< 0.001*		< 0.001*		0.077		< 0.001*	

*Note:* +, number of positive samples; −, number of negative samples. The chi‐square (χ2) test was performed using SPSS. A *p* < 0.05 is indicated by * and is considered statistically significant (in bold). Spring, 04–06; Summer, 07–09; Autumn, 10–12; Winter, 01–03.

### 3.4 Coinfection

There was no significant difference in the coinfection rate between males and females. However, a significant difference in the coinfection rate was observed among the four age groups (*p* < 0.05). The adolescent group had the lowest rate of multiple infections, at 6.36% (37/582), whereas the other three groups exhibited infection rates ranging from 10.33% to 12.72%. Additionally, the coinfection rate among inpatients was 13.81% (152/1101), which was significantly higher than that among outpatients, which was 4.80% (33/687) (*p* < 0.05) (Table 3).

**TABLE 3:** Characteristics of children with coinfection.

Characteristic	Single infection (*N* = 854)	Coinfection (*N* = 185)	Coinfection rate
Sex			
Male	501	114	10.61% (114/1074)
Female	353	71	9.94% (71/714)
*χ2*	0.550		
*p*	0.458		
Age group			
< 1 year	107	22	10.33% (22/213)
1 to < 3 years	203	53	12.65% (53/419)
3 to < 5 years	272	73	12.72% (73/574)
5 to < 18 years	272	37	6.36% (37/582)
*χ2*	**11.350**		
*p*	**< 0.05**		
Case type			
Inpatients	531	152	13.81% (152/1101)
Outpatients	323	33	4.80% (33/687)
*χ2*	**26.962**		
*p*	**< 0.001**		
All	854	185	

*Note:* The chi‐square (χ2) test was performed using SPSS. A *p* < 0.05 is indicated by * and is considered statistically significant (in bold).

**FIGURE 5:**
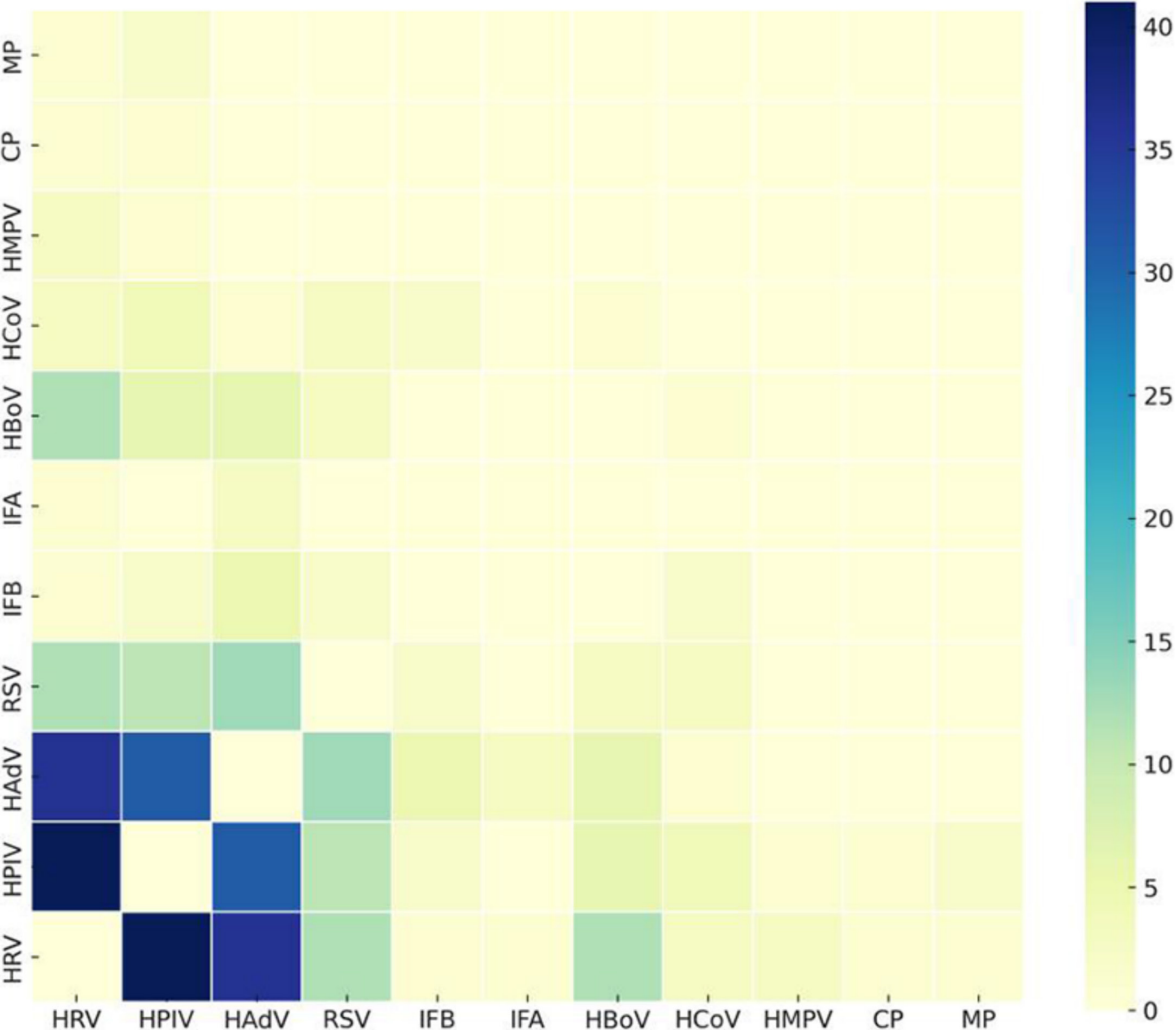
Coinfection pattern of pathogens in patients with fever.

The most common infection combinations included HRV combined with HAdV and HPIV, followed by HPIV combined with HAdV (Figure 5).

## 4 Discussion

RTIs are a frequent reason for pediatric outpatient visits and remain a significant cause of mortality among children under the age of 5. Among these infections, viruses are the predominant culprits, warranting close attention from pediatricians.

Between November 2020 and June 2022, we conducted an investigation into the prevalence of pathogens in 1788 pediatric patients (under 18 years of age) presenting with fever symptoms in Guangdong. Our findings revealed that 58.1% of these patients were infected with at least one pathogen. This proportion is close to many previous domestic studies conducted prior to the COVID‐19 pandemic [15, 16]. Our data collection was primarily concentrated in 2021. Following the outbreak of the new coronavirus at the end of 2019, lockdown measures were implemented, resulting in fewer patients seeking hospital treatment, particularly those with more severe clinical symptoms. Additionally, the pandemic control measures may have influenced the population's immunity to respiratory viruses. A Taiwanese study demonstrated a significant decrease in the positive rate for multiple respiratory viruses from early 2020 until the latter half of 2020 [17]. Another Chinese study reported a reduction in both sample numbers and positivity rates for multiple pathogens from the onset of the 2019 epidemic through the end of 2020 [18]. In light of these factors, the 58.1% positivity rate observed in our study reflects the complex interplay of decreased healthcare‐seeking behavior and altered virus exposure during the pandemic. Thus, this rate is considered appropriate and informative given the exceptional circumstances of our sampling period.

HRV, HPIV, HAdV, and RSV were the most frequently detected pathogens in febrile pediatric patients. The positive rates of HRV were quite consistent across the four age groups, ranging from 17.3% to 20.3%. HRV is recognized as one of the common respiratory viral pathogens affecting both adults and children [19], and our findings align with this observation. HPIV and HAdV are also widely prevalent respiratory viruses. HPIV tends to affect children under 5 years of age and elderly individuals over 60 years old more frequently, whereas HAdV infects individuals across all age groups [2, 20]. Our data mirrors these age‐specific trends. Notably, RSV stands out as the predominant pathogen responsible for lower respiratory tract infections in infants and young children. A global review report highlights the substantial disease burden associated with RSV, affecting one in every 50 children aged 0–60 months and one in every 28 children aged 28 days. Moreover, one child out of every 6 months old succumbs to RSV [21]. In our dataset, RSV positivity was most pronounced among children less than 1 year old.

We observed variations in the prevalence spectrum of pathogens between outpatient and inpatient cases. HRV and HPIV exhibited twice the positive rate among inpatients compared to outpatients, whereas influenza viruses (IFA + IFB) had a positive rate over three times higher in outpatients than in inpatients. Analysis of the seasonal patterns of these pathogens revealed an interesting contrast with RSV, which showed a peak prevalence during the summer months, in contrast to the seasonal trends of other pathogens. Prior to the COVID‐19 outbreak, RSV detection rates were typically higher during winter and lower during summer [4, 15], However, following the COVID‐19 outbreak, RSV detection rates declined significantly worldwide. Subsequently, as prevention and control measures were relaxed and schools reopened, RSV experienced an out‐of‐season epidemics [22]. A longitudinal study tracking RSV activity from 2017 to 2023 revealed: The customary winter RSV epidemic was absent in the 2020–2021 season, and the epidemic for 2021–2022 commenced unusually in spring, peaked in summer; the 2022–23 season started and peaked later than the 2021–2022 season, but earlier than pre‐pandemic seasons [23].

In addition to changes in the pathogenic spectrum, the phenomenon of coinfections also warrants attention. Coinfection with respiratory viruses occurs when an individual is infected by two or more viruses simultaneously. The relationship between coinfections and clinical symptoms has yielded varied conclusions, and the underlying mechanisms remain to be elucidated. A review from 2020 suggested that coinfections among viruses may not necessarily increase the disease burden, whereas coinfections involving both bacteria and viruses might [24]. Surveillance data from the Chinese National Center for Disease Control and Prevention spanning from 2009 to 2019 indicated that coinfections among viruses generally exhibit negative interactions that inhibit each other's growth, but interactions between viruses and bacteria have a positive effect [2]. Our own data reveals significant differences in co‐detection rates among different age groups and patient categories. Similar to the positivity rate patterns, children under 5 years of age and hospitalized patients tend to exhibit higher co‐detection rates, with correspondingly elevated positivity rates in these groups. The predominant infection combinations observed in our study are HRV combined with HAdV and HPIV, followed by HPIV combined with HAdV. These pathogens also exhibit high infection rates individually. Although this coinfection combination aligns with statistical trends, it diverges from the combination patterns observed by the National Center for Disease Control and Prevention. The epidemiological characteristics of coinfections and their clinical implications require further research. Our study is subject to several limitations, including the absence of comprehensive patient clinical data and corresponding clinical feature analysis. Additionally, for certain pathogens such as HPIV and HCoV, we did not differentiate between subtypes.

## 5 Conclusion

In summary, our epidemiological investigation yielded the following findings:

- Distinct pathogen prevalence spectra were identified across different age groups, as well as between inpatients and outpatients. Children under the age of 5 and inpatients have a higher likelihood of experiencing multiple infections, necessitating the testing of various pathogens. In cases where testing for all 11 pathogens is not feasible, that two groups should at least be tested for RSV, HRV, HPIV, and HAdV.
- RSV exhibited counter‐seasonal circulation, deviating from the typical seasonal prevalence trends of other pathogens. Following the epidemic, RSV could undergo further changes, underscoring the need for persistent monitoring.
- Coinfections were detected among several pathogens with the highest positive rates. The prevalence spectrum of respiratory pathogens proves to be intricate and uncertain, underscoring the importance of employing nucleic acid detection reagents for multiple pathogens in screening tests.

## Author Contributions

Qianwen Zhao and Peifeng Ke are the guarantors of the entire study. Qianwen Zhao and Lingxiao Jiang designed this study. Qianwen Zhao and Peifeng Ke did the literature research. Qianwen Zhao and Peifeng Ke received the funding support. Lingshan Hu, Changhong Jiang, Rong Su, Weifeng Lv, Qixin Li, Lingxiao Jiang, and Donglin Cao acquired the data. Lingxiao Jiang and Donglin Cao drafted the paper. Qianwen Zhao and Peifeng Ke revised the paper. All authors have read and approved the final paper.

## Ethics Statement

All procedures performed in studies involving human participants were in accordance with the ethical standards of the institutional and/or national research committee and with the 1964 Helsinki declaration and its later amendments or comparable ethical standards. This study was approved by the Ethics Committee of Zhujiang Hospital, Southern Medical University and written informed consent was obtained from the patient or their parents.

## Conflicts of Interest

The authors declare no conflicts of interest.

## Data Availability

All data included in this study are available upon request by contact with the corresponding author.
